# FEM simulation of breast deformation with semi-fluid representation

**DOI:** 10.1007/s11548-024-03288-8

**Published:** 2024-12-16

**Authors:** Shota Takahashi, Hiroshi Fujimoto, Katsuhiro Nasu, Toshiya Nakaguchi, Naoto Ienaga, Yoshihiro Kuroda

**Affiliations:** 1 Degree Programs in System and Information Engineering, University of Tsukuba, 1-1-1 Tennodai, Tsukuba City, Ibaraki 305-8573 Japan; 2 Department of General Surgery, Chiba University Graduate School of Medicine, 1-8-1 Inohana Chuo-ku, Chiba City, Chiba 260-0856 Japan; 3 Comprehensive Radiology Center, Chiba University Hospital, 1-8-1 Inohana Chuo-ku, Chiba City, Chiba 266-8677 Japan; 4 Center for Frontier Medical Engineering, Chiba University, 1-33 Yayoi Cho Inage Ku, Chiba City, Chiba 263-8522 Japan; 5 Institute of Systems and Information Engineering, University of Tsukuba, 1-1-1 Tennodai, Tsukuba City, Ibaraki 305-8573 Japan

**Keywords:** Image-guided surgery, Magnetic resonance imaging, Finite element method, Semi-fluidity, Anchoring structure

## Abstract

**Purpose:**

In image-guided surgery for breast cancer, the representation of the breast deformation between planning and surgery plays a key role. The breast deforms significantly and behaves as a fluid with some constraints. Concretely, the deep fat layer in the breast deforms fluidly due to its incomplete fixation to the chest wall, while the anchoring structures by fascia avoid excessive deformation. In this study, we propose a method to simulate the semi-fluid deformation of the breast, considering the fluidic properties of the adipose tissue under the constraints of the anchoring structures.

**Methods:**

The proposed method prioritizes anatomical features of the breast, enhancing tissue mobility near the chest wall and modeling the anchoring structure of the fascia along the inframammary fold. To simulate semi-fluid deformation, constraint force from anchoring structure is applied to prone-positioned breast model, using a finite element method.

**Results:**

The results of the evaluation indicate a tumor center registration error of 11.87 ± 4.05 mm. Additionally, we verified how semi-fluid representation affects the registration error. The tumor’s Hausdorff distance decreased from 12.89 ± 6.24 mm to 11.50 ± 4.38 mm with considering semi-fluidity.

**Conclusion:**

The results showed that the use of semi-fluid representation tends to reduce registration errors. Therefore, it was suggested that the proposed method could improve the accuracy of breast posture conversion.

## Introduction

**Fig. 1:**
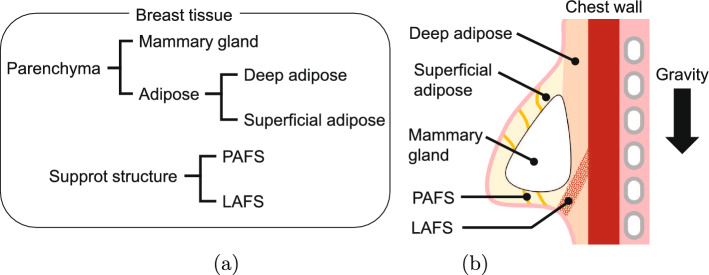
**a** Hierarchical classification of breast tissue. **b** Anatomical structure of breast. The arrow shows the direction of gravity

**Fig. 2:**
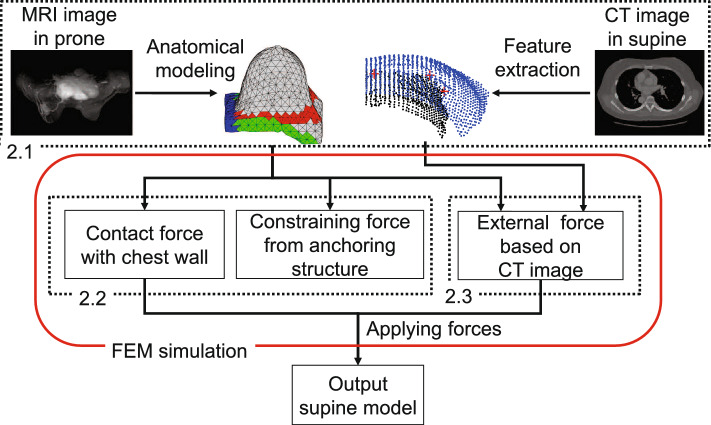
Flow of the proposed method

To ensure the precise removal of the lesion of breast cancer, which is common in women, augmented reality assisted surgery is being developed [1, 2]. This involves projecting magnetic resonance (MR) images, taken in the supine position, onto the patient’s body surface during breast cancer surgery [3]. However, preoperative MR images are usually acquired in the prone position to observe the breast in the stretched position. Therefore, there is a clinical demand to transform these images to the surgical supine position. The classification of the tissues that make up the breast and the anatomical structure are shown in Fig. 1. The adipose tissue of the breast consists of a deep adipose and superficial adipose. Within those adipose, there are supporting structures known as the Protective AdipoFascial System (PAFS) and the Lubricant AdipoFascial System (LAFS), which are fascia. The PAFS, which is located superficially, strongly supports the glandular-skin interface and facilitates coordinated movement of the glandular, adipose, and skin tissues. In contrast, the LAFS is located at the boundary between the glandular tissue and the chest wall and provides mobility to the adipose tissue, resulting in a fluid deformation of the entire breast [4, 5]. In addition, the LAFS is referred to as an anchoring structure because the loose support of the inframammary fold provides a constraint on fluid deformation [6].

With regard to studies attempting to deform the breast from the prone to supine position, physical simulations using a finite element method (FEM) based on continuum mechanics have been widely used due to the challenges of non-physical constraints in non-linear registration [7–11]. It is difficult to obtain material parameters such as Young’s modulus and Poisson’s ratio for each tissue in biological simulations. Therefore, the linearized iterative boundary reconstruction (LIBR) method has been developed to estimate unknown deformations based on known deformations of the surface and feature points whose location information is known  [10, 12]. However, in conventional simulations of breast deformation from the prone to supine position, the chest wall has been treated as a fixed boundary condition [13–15]. Some studies do not fix the chest wall in mammography compression simulations [16], or only consider fat fluidity [17], but no studies have explicitly expressed semi-fluidity.

The objective of this study is to simulate the semi-fluid deformation of the breast in order to convert a prone breast model to the supine position. The proposed method uses finite element simulation. In addition to using supine computed tomography (CT) images as known deformation information for the LIBR method, it also represents the semi-fluid deformation due to the fluidity of adipose tissue and the constraint forces caused by fascia. The engineering contributions of this study are as follows:

- Proposal of a posture conversion method for the finite element breast model using semi-fluid representation
- Examining the impact of semi-fluid representations on the resultant deformation.

**Fig. 3:**
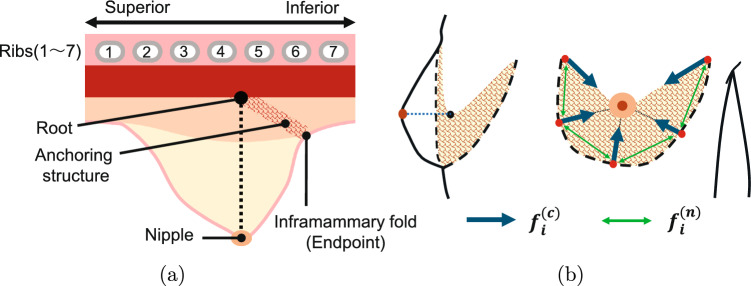
**a** Positions of the nipple, anchoring structure, and its root and endpoint in the Sagittal plane. **b** Constraint forces from membranous fascia. The black dashed line is the deep fascia endpoint, along the inframammary fold, from the mid-chest to the axilla

## Method

### Modeling of anatomical structures

The processing flow of the proposed method is shown in Fig. 2. A tetrahedral mesh model is created from the prone breast MR images. In this study, we employed a linear elastic model as the mechanical model because we have extended the linearized iterative boundary reconstruction (LIBR) method [12], which also applies a linear elastic model, to correspond the model to the measured geometry. The midline of the chest is set as a fixed boundary condition. From the supine CT image, we obtain point clouds of the breast surface and chest wall, as well as the position coordinates of the nipple and vessel bifurcation points. To express the fluidity of the adipose tissue, the Young’s modulus of the elements corresponding to the deep adipose tissue within 15 mm thick from the chest wall surface is reduced to increase the fluidity. The thickness of the deep adipose tissue was determined by visually measuring the distance between the chest wall and the mammary gland in all cases and averaging the results. The superficial adipose tissue at the border of the skin and glandular tissue has the same Young’s modulus as the glandular tissue because it is connected to the glandular tissue by PAFS. For LAFS, the root is near the intersection of the vertical lines from the nipple of the supine breast down to the chest wall, and the end point corresponds to the inframammary fold, the boundary between the breast and the surrounding skin. According to previous study, the deep fascia is present between the mid-chest and the axilla along the inframammary line of the breast [6]. Therefore, this region was used as the end point for the constraining forces. The positional relationships of the support structure, its root, and end points are shown in Fig. 3a.

### Semi-fluid representation

The displacement ${\varvec{u}}$ of the prone breast model is determined based on FEM and is given by the following equation:

1 $$\begin{aligned} K {\varvec{u}} = {\varvec{F_\mathrm{{g}}}} + {\varvec{F_\mathrm{{p}}}} + {\varvec{F_\mathrm{{a}}}} + {\varvec{[f_1 f_2 \cdots f_m] \alpha }} \end{aligned}$$

where ${\varvec{F_\mathrm{{g}}}}$ is the gravitational force due to the volume of each element, ${\varvec{F_\mathrm{{p}}}}$ is the contact force with the chest wall, ${\varvec{F_\mathrm{{a}}}}$ is the restraint force due to the support structure, and ${\varvec{[f_1 f_2 \cdots f_m] \alpha }}$ is an external force based on observation.

The contact force with the chest wall is calculated using a penalty method. First, a second order approximate surface is expressed from the supine chest wall point cloud. The expressed approximate surface equation is used to determine the penetration into the chest wall surface, and then the external force is penalized by the weight of the penetration distance. The contact force ${\varvec{f^{(p)}_i}}$ at the *i* th node is expressed by the following equation.

2 $$\begin{aligned} {\varvec{f^{(p)}_i}} = w^{(p)}{\varvec{E_i}} \end{aligned}$$

where ${\varvec{E_i}}$ is the penetration distance into the approximate surface, and $w^{(p)}$ is the weight for the penetration distance. The total load vector ${\varvec{f^{(p)}_i}}$ is the sum of the contact forces with the chest wall ${\varvec{F_\mathrm{{p}}}}$ at all nodes.

The constraint forces due to the anchoring structure are expressed by applying a force between the root and the endpoints, and between the adjacent endpoints. This is a membranous extension of the constraint by ligaments, where the constraint is expressed in one direction only [18, 19]. The constraint force ${\varvec{f^{(a)}_i}}$ at the *i* th node is expressed by the following equation using the constraint force ${\varvec{f^{(c)}_i}}$ in the root-to-endpoint direction and the constraint force ${\varvec{f^{(n)}_i}}$ in the adjacent node direction.

3 $$\begin{aligned}  &   {\varvec{f^{(a)}_{i}}} = {\varvec{f^{(c)}_{i}}} + \sum _j {\varvec{f^{(n)}_{i,j}}} \end{aligned}$$

4 $$\begin{aligned}  &   {\varvec{f^{(c)}_{i}}} = {\left\{ \begin{array}{ll} {\varvec{0}} &  (|{\varvec{q_i}}| < u^{(0)}_i)\\ w^{(a)} \frac{(|{\varvec{q_i}}| - u^{(0)}_i)}{u^{(0)}_i|{\varvec{q_i}}|}{\varvec{q_i}}& (|{\varvec{q_i}}| \ge u^{(0)}_i)\\ \end{array}\right. } \end{aligned}$$

5 $$\begin{aligned}  &   {\varvec{f^{(n)}_{i,j}}} = {\left\{ \begin{array}{ll} {\varvec{0}} &  (|{\varvec{s_{i,j}}}|< |{\varvec{s^{(0)}_{i,j}}}|)\\ w^{(a)} \frac{|{\varvec{s_{i,j}}}| - |{\varvec{s^{(0)}_{i,j}}}|}{|{\varvec{s^{(0)}_{i,j}}}|{\varvec{q_i}}|}{\varvec{q_i}}& (|{\varvec{s_{i,j}}}| < |{\varvec{s^{(0)}_{i,j}}}|)\\ \end{array}\right. } \end{aligned}$$

where ${\varvec{q_i}}$ is the direction vector of the constraining force, from the root of the support structure to the end points, $u^{(0)}_i$ is the size of ${\varvec{q_i}}$ in the initial state model. ${\varvec{s_{i,j}}}$ is the direction vector to the *j*-th node adjacent to the *i* th node, ${\varvec{s^{(0)}_{i,j}}}$ is the initial value of ${\varvec{s_{i,j}}}$. $w^{(a)}$ is the weight of the support force, and the constraint force is applied according to the magnitude above a certain threshold value. Let ${\varvec{F^{(a)}_i}}$ be the total load vector summed at all nodes and ${\varvec{F_\mathrm{{a}}}}$ be the constraint force due to the support structure. The location of the constraint forces in the breast is shown in Fig. 3b.

### External force based on CT image

The LIBR method [12] is used to minimize alignment errors with the supine CT image. The LIBR method represents any loads applied to the model by a linear combination of deformation bases, which are weights used to drive the model. The error between the supine CT image and the prone model is formulated for each of the three features: surface, chest wall, and fiducial points. The surface and chest wall errors are calculated as the Euclidean distance between the point cloud data points and the polygons of the prone model, while the fiducial point errors are calculated as the Euclidean distance between the 3D positions. By summing the squares of the errors in all calculated features, the objective function $F({\varvec{\alpha }})$ is obtained with ${\varvec{\alpha }}$ as the variable. Then, $\tilde{{\varvec{\alpha }}}$, which is defined as ${\varvec{\alpha }}$ that minimizes $F({\varvec{\alpha }})$, is the optimal solution of ${\varvec{\alpha }}$.

6 $$\begin{aligned}  &   F({\varvec{\alpha }}) = \sum _{i=1}^{N_\textrm{skin}} ((S_i\hat{{\varvec{n}}}) \cdot {\varvec{p}}_i({\varvec{\alpha }}))^2 + \sum _{i=1}^{N_\textrm{chest}} ((S_i\hat{{\varvec{n}}}) \cdot {\varvec{p}}_i({\varvec{\alpha }}))^2 \nonumber \\  &   \qquad \qquad + \sum _{i=1}^{N_\textrm{fiducial}} {p}_i({\varvec{\alpha }})^2 \nonumber \\  &   \qquad \qquad + \lambda _1 |{\varvec{\alpha }}| + \lambda _2 \sum _{i = 1}^{N_\textrm{surface}} K_i \end{aligned}$$

7 $$\begin{aligned}  &   \tilde{{\varvec{\alpha }}} = {\mathop {\textrm{argmin}}\limits _{{\varvec{\alpha }}}}\, F({\varvec{\alpha }}) \end{aligned}$$

where $N_\textrm{skin}$, $N_\textrm{chest}$, and $N_\textrm{vessel}$ are the number of data points at the surface, chest wall and fiducial point, respectively. ${\varvec{p}}_i({\varvec{\alpha }})$ is the direction vector from a node on the model to the *i*th data point in the point cloud. $S_i\hat{{\varvec{n}}}$ is the normal vector closest to a data point. The $\lambda _1, \lambda _2$ are the regularization parameters for the magnitude of the weight ${\varvec{\alpha }}$ and the surface smoothness $K_i$, respectively. $K_i$ is calculated as the sum of the inner product of the normal vector at each node and the surrounding polygons. The optimization algorithm used is the Levenberg-Marquardt method [20], which is suitable for nonlinear least squares optimization problems.

## Result

### Data preprocessing

**Fig. 4:**
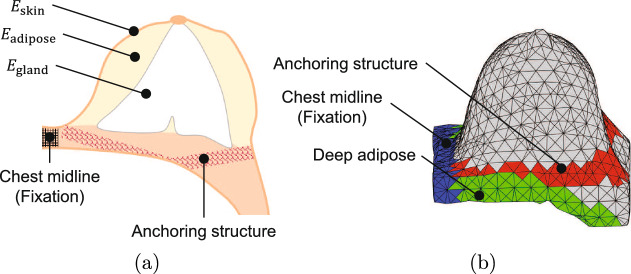
**a** The correspondence between the Young’s modulus and position for each region. **b** Created breast mesh and components

To evaluate the proposed method, we used nine cases of both prone MR images and supine CT images. The local ethics committee approved this study (Chiba University Hospital on October 23, 2020 No. 3906). No additional CT scan is required. Preoperative CT images were obtained to precisely ascertain tumor location and screen for axillary involvement and distant metastases. In our internal criteria, the patients underwent CT scans as a component of their preoperative work-out, that is for clinical, and not for research, purposes. In all cases, distinct tumor lesions with clear boundaries were observed. Segmentation was performed semi-automatically using the region growing method implemented in 3D Slicer [21], a well-known image computing platform. For both MR and CT images, the boundary between the breast and chest wall was visually inspected on several slices. After manually segmenting a few slices, semi-automatic segmentation was carried out across the entire set of slices using interpolation. For the constraint endpoints, the vertices of the created mesh were specified manually using MeshLab [22], based on the criteria described in “Modeling of anatomical structures” section. The model parameters are listed in Table 1. The correspondence between the Young’s modulus and the location of each area is illustrated in Fig. 4. In the MRI and CT images, radiologists visually identified and measured the 3D positions of the following six characteristic points and established correspondences between them.

- Right sternoclavicular joint
- Left sternoclavicular joint.
- Lower sternum
- Nipple on the affected side
- Vascular bifurcation on the affected side (1)
- Vascular bifurcation on the affected side (2)

Alignment between MR and CT images was performed by obtaining translation and rotation matrices from three points: the right sternoclavicular joint, the left sternoclavicular joint, and the lower end of the sternum. The nipple of the affected side and two vascular bifurcations were used as anatomical fiducial points.

**Table 1:** Parameters in the simulation

	Parameter	Value
Breast model	Num. of nodes	820~2695
	Num. of elements	2888~10760
	$E_\textrm{skin}$ [kPa]	4
	$E_\textrm{adipose}$ [kPa]	9.75
	$E_\textrm{gland}$ [kPa]	9.75
	Poisson’s ratio	0.495
Simulation parameters	Num. of control points	70
	$\lambda _1$	0.01
	$\lambda _2$	0.6
	$\lambda _k$	10

**Fig. 5:**
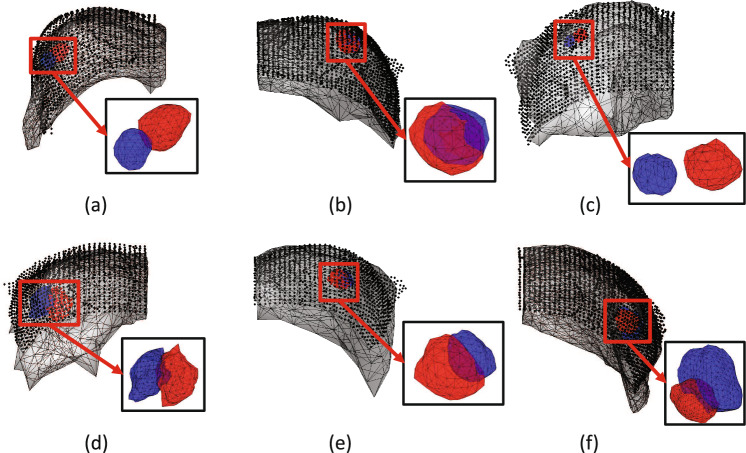
**a**–**c**: The best cases from each group in the cross-validation. **d**–**f**: The worst cases from each group in the cross-validation. The deformed model, surface data, estimated tumor position, and tumor ground truth are colored gray, black, red, and blue, respectively

### Calculation of optimal parameters

The optimal values for the parameters related to the semi-fluid representation, namely the weight of the support force $w^{(a)}$ and $E^{(a)}$, the Young’s modulus of the deep adipose, were determined by running simulations with different values and verifying the accuracy of the tumor position alignment. The weight of the support force was empirically validated with six values: 0.00003, 0.00006, 0.00012, 0.00018, 0.00024, and 0.0003. The Young’s modulus of the deep adipose tissue was validated with five values: 0.10 kPa, 0.50 kPa, 1.0 kPa, 1.5 kPa, and 2.0 kPa, following the work of Dufaye et al. [23]. The nine cases were divided into six for training and three for validation. For each of the six training datasets, we ran simulations with varying parameters. The optimal values were determined as those that minimized the average tumor center alignment error in the training data. The tumor center was calculated as follows:

8 $$\begin{aligned} centroid=\left| \left| \frac{1}{N_p}\sum ^{N_p}_{i}{\varvec{x}}_{\textrm{p},i} - \frac{1}{N_s}\sum ^{N_s}_{j}{\varvec{x}}_{\textrm{s},j}\right| \right| \end{aligned}$$

where $N_p$ is the number of estimated prone tumor point clouds, ${\varvec{x}}_{\textrm{p},i}$ is the *i*th data point in the prone tumor point group $X_{p}$, $N_s$ is the number of supine tumor point clouds that are ground truth, and ${\varvec{x}}_{\textrm{s},j}$ is the *j*th data point in the supine tumor point group $X_{s}$. Using the determined optimal parameters, simulations were performed on the validation data. Cross-validation was used to calculate the final accuracy of the proposed method.

Of the validation results for each of the groups of cv1~cv3 created during the cross-validation, the most accurate cases are shown in Fig. 5**a**–**c**, the least accurate cases are shown in **d**–**f**. As shown in Table 2, the cross-validation results showed that the optimal parameters for all data were 0.0001 for $w^{(a)}$ and 0.83 for $E^{(a)}$. These values are used for statistical validation.

**Table 2:** Obtained optimal parameters for cross validation

	Best $w^{(a)}$	Best $E^{(a)}$	Error (mm)
cv1	0.00012	1.5	$15.01 \pm 2.23$
cv2	0.00012	0.5	$8.26 \pm 4.66$
cv3	0.00006	0.5	$13.88 \pm 3.61$
Mean	0.0001	0.83	$11.87 \pm 4.05$

**Table 3:** Hausdorff distance for each comparison method

	Chest wall movement	Fluidity	Anchoring	Hausdorff distance (mm)
(A)	–	–	–	25.98 ± 11.63
(B)	$\checkmark$	–	–	14.36 ± 7.74
(C)	$\checkmark$	$\checkmark$	–	12.89 ± 6.24
Proposed	$\checkmark$	$\checkmark$	$\checkmark$	11.50 ± 4.38

**Fig. 6:**
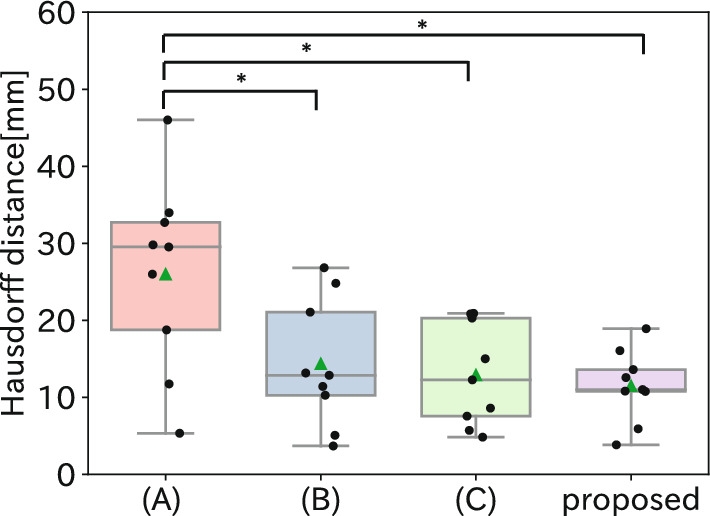
Boxplot of the Hausdorff distance for all comparison methods and the proposed method. Black circles and green triangles represent individual cases and average distances, respectively. *Indicates $p<$ 0.05

### Validating the effect of the semi-fluid representation

To test the validity of the semi-fluid representation, we tested how chest wall fixation, deep adipose fluidity, and support structure constraint affect the results. In the proposed method, the chest wall is not fixed, the mobility is increased by reducing the Young’s modulus of the deep adipose, and the semi-fluidity is represented by modeling the constraint force by the support structure according to Eq. 4. The following three methods (A)~(C) were compared with the proposed method.

- (A): No chest wall movement + no fluidity + no anchoring force
- (B): Chest wall movement + no fluidity + no anchoring force
- (C): Chest wall movement + fluidity + no anchoring force
- Proposed method: Chest wall movement + fluidity + anchoring force

In the case of no fluidity, the Young’s modulus $E^{(a)}$ of the deep adipose is set to 9.75 kPa, the same as that of the mammary gland, to limit the fluidity. In the case of no support, the simulation is performed with $w^{(a)}$, the support weight, set to 0. The Hausdorff distance was used as the evaluation index. The Hausdorff distance indicates the extent to which the tumor can be removed with a margin during surgery. In this study, the Hausdorff distance between the estimated tumor in prone position and the ground truth tumor in supine position was calculated as follows:

9 $$\begin{aligned} hausdorff = \max _{{\varvec{x}}_{\textrm{s,j}} \in X_{s}}(\min _{{\varvec{x}}_{\textrm{p,i}} \in X_{p}}(||{\varvec{x}}_{\textrm{s},j} - {\varvec{x}}_{\textrm{p},i}||)). \end{aligned}$$

The distribution of Hausdorff distances for each method is shown in Fig. 6, and the mean Hausdorff distance for each method is shown in Table 3. F-test was performed on the results, and then the null hypothesis was rejected, so equal variances cannot be assumed. Next, the Friedman test was performed and multiple comparisons were made using the Wilcoxon signed rank test with Bonferroni correction. The test results show a significant difference between the method (A) and the other methods including the proposed method. The significant difference in accuracy between the method (A) and the proposed method demonstrated the effectiveness of not fixing the chest wall and taking into account the movement of deep adipose. No statistically significant differences were found between the proposed method and methods (B) and (C), but there was a trend toward smaller Hausdorff distances.

## Conclusion and future work

In this study, a method was developed to convert the prone breast model to the supine position by focusing on the semi-fluidity of the breast tissue. The results showed that the consideration of semi-fluidity tended to improve the accuracy of the method. This study has some limitations. First, the range of fluidic adipose tissue was set to 15 mm in all cases. However, there are individual differences in the distance between the chest wall and the glandular tissue. Since the boundary between glandular and adipose tissue is easily captured as an image feature in MR images, it is possible to set the distance of the boundary individually for each case. Second, regarding the mechanical properties model used in this study, we employed a linear elastic model. However, using the Neo-Hookean model, a nonlinear elastic model, could potentially improve accuracy, as it is more effective in representing the large deformations of the breast [15]. Third is the pre-stress conditions. In the initial prone position, gravity is applied and stress is generated. Therefore, it is necessary to perform actions such as removing the load caused by gravity, as was done in previous studies [24]. In this study, the nonlinear dynamics of the breast, which are in fact complex, are simplified and simulated. For example, the fascia, which is an anchoring structure, is not actually homogeneous and varies in strength depending on whether it is medial or lateral to the body center, upper or lower. In addition, mammary gland density, which changes with age, and collagen to elastin composition ratio affect individual differences in breast deformation. There are three main types of chest wall contours: A-frame roof, burrel chest, and flat rectangular chest. These are the factors that cause individual differences in breast deformity. In the less common cases, pectus excavatum, contact simulation may be necessary because of the possibility of contact between the left and right breasts. There is a fascial cleft between the Cooper’s ligament, and the deep fascia, which moves independently between the two layers but is treated as the same elastic body in this study. For more accurate modeling and application to real patients, these variables need to be considered and properly represented as much as possible.
